# Supplementary material for: MetaRibo-Seq measures translation in microbiomes
Source: Nat Commun. 2020 Jun 29;11:3268. doi: 10.1038/s41467-020-17081-z (PMC7324362; doi:10.1038/s41467-020-17081-z)
Supplement: Supplementary file 10 — Supplementary Data 7 [file 41467_2020_17081_MOESM10_ESM.zip › File2/Confidence_High_Taxonomy/416619_out.krona.html]

Javascript must be enabled to view this page.

members
magnitude
magnitudeUnassigned
count
unassigned
taxon
rank

416619\_out

3

3
superkingdom
2

3
1239
phylum

2
species
1263007

SRS015579\_contig\_number\_19951SRS098881\_contig\_number\_contig-100\_24508.24509

186801
class
1

1
186802
order

1
family
541000

946234
genus
1

species
1193534

SRS049402\_contig\_number\_9993
1
